# Hospital Meetings

**Published:** 1906-05-05

**Authors:** 


### HOSPITAL MEETINGS.

### EVELINA HOSPITAL FOR SICK CHILDREN.

At the Annual Court of Governors on April 27 the chair was taken by Mr. William Aste, in the absence of the Earl of Ancaster, the President. The Chairman, in moving the adoption of the report and balance-sheet for 1905, said that they would notice that the financial condition of the hospital during the past year had not been very good. They had ended the year with a deficit of £1,215, which was not at all satisfactory. They earnestly desired to get more annual subscriptions—there had been a falling-off under this head last year of £40. He trusted they would all try and get as many as possible to take an interest in the hospital, especially in view of the important matter which was facing them—namely, the reconstruction of the out-patient department. King Edward's Hospital Fund and the Hospital Sunday Fund had been pointing out to them for some time the inadequacy of their present arrangements, and the committee were determined at all hazards to take the work in hand. It was rather a big order, since it would cost at least £12,000; as yet they had only £1,600, but the Council of King Edward's Hospital Fund had promised substantial help when the work was once begun. The committee wished to express their thankfulness to the trustees who, by reinvesting much of the property of the hospital, had increased the income by about £350 per annum. They were also very grateful to the kind friend by whose generosity a certain number of cots were reserved at convalescent homes for cases from their hospital. This was an immense advantage to the work of the institution, as many cases that required long treatment could now be sent to the homes, where every care and attention was given them, and the beds at the hospital were much eased. He concluded with an urgent appeal to help on the good work of the hospital by endeavouring to get new subscribers.

The motion was seconded by Mr. Malcolm Scott, who remarked that an idea had gone abroad that because the late Baron Ferdinand Rothschild was their founder they had the Rothschild family at their back. This was not the case, though they had always been most generous, the hospital being entirely dependent on its endowment and on the annual subscriptions. The work had been begun on a small scale, but had grown rapidly and the requirements for medical and surgical work increased every year.

The report having been adopted, various votes of thanks were passed, including one to the Chairman for presiding.

